# Concerned significant others of people with gambling problems in Finland: a cross-sectional population study

**DOI:** 10.1186/1471-2458-14-398

**Published:** 2014-04-24

**Authors:** Anne H Salonen, Sari Castrén, Hannu Alho, Tuuli Lahti

**Affiliations:** 1 National Institute for Health and Welfare, Department of Mental Health and Substance Abuse Services, P.O. Box 30, Helsinki FIN-00271, Finland; 2 Research Unit of Substance Abuse Medicine, University of Helsinki, Helsinki, Finland; 3 Faculty of Social Sciences, Department of Behavioral Sciences and Philosophy, University of Turku, Turku, Finland

**Keywords:** Concerned significant others, Cross-sectional, Population study, Problem gambling

## Abstract

**Background:**

Problem gambling not only impacts those directly involved, but also the concerned significant others (CSOs) of problem gamblers. The aims of this study were to investigate the proportion of male and female CSOs at the population level; to investigate who the CSOs were concerned about; and to investigate sociodemographic factors, gender differences, gambling behaviour, and health and well-being among CSOs and non-CSOs.

**Methods:**

The data (n = 4484) were based on a cross-sectional population study. Structured telephone interviews were conducted in 2011–2012. The data were weighted based on age, gender and residency. The respondents were defined as CSOs if they reported that at least one of their significant others (father, mother, sister/brother, grandparent, spouse, own child/children, close friend) had had gambling problems. Statistical significance was determined by chi-squared and Fisher’s exact tests, and logistic regression analysis.

**Results:**

Altogether, 19.3% of the respondents were identified as CSOs. Most commonly, the problem gambler was a close friend (12.4%) of the CSO. The percentage of close friends having a gambling problem was larger among male CSOs (14.4%) compared with female CSOs (10.3%; p ≤ 0.001), while the percentage of partners with gambling problem was larger among females (2.6%) than among males (0.8%; p ≤ 0.001). In the best fitting model, the odds ratio (95% CI) of being a male CSO was 2.03 (1.24–3.31) for past-year gambling problems, 1.46 (1.08–1.97) for loneliness and 1.78 (1.38–2.29) for risky alcohol consumption. The odds ratio (95% CI) of being a female CSO was 1.51 (1.09–2.08) for past-year gambling involvement, 3.05 (1.18-7.90) for past-year gambling problems, 2.21 (1.24–3.93) for mental health problems, 1.39 (1.03–1.89) for loneliness and 1.97 (1.43–2.71) for daily smoking.

**Conclusions:**

CSOs of problem gamblers often experience cumulating problems such as their own risky gambling behaviour, health problems and other addictive disorders. The clearest gender difference was seen in smoking by CSO. In order to develop efficient and targeted support and services for CSOs, it is necessary to understand the correlates related to different subgroups of CSOs.

## Background

At the population level, estimated problem-gambling prevalence rates vary between countries, from 0.2 to 5.3% [1]. However, problem gambling not only impacts those directly involved, but also a variety of their significant others [2-4]. The destructive effects of problem gambling include not only significant financial problems, but also emotional, relationship and social problems [5-8]. There is also evidence of gender differences, which should be taken into account in prevention and developing support for gambling problems [9].

Concerned significant others (CSOs) refer to people in the surrounding environment of a person who has gambling problems [2,3,9]. The broadness of different definitions for CSOs varies: the CSOs of the person with gambling problems can be parents, spouses, a boyfriend/girlfriend, own children or any other family members or relatives, or more distant friends or colleagues [9]. It has been proposed that each gambler’s gambling problem has destructive effects on as many as 7 to 16 other people [10,11].

Previously, only two peer-reviewed research articles have examined CSOs from the epidemiological perspective. In Norway, Wentzel and colleagues (2008) examined CSOs within the family context using two questions based on the Lie/Bet instrument and identified 2.0% of the population as CSOs [3]. In Sweden, Svensson and colleagues [2013] studied CSOs by using a somewhat wider and more open approach and identified 18.2% of the population as CSOs [9]. However, the Swedish study did not define how the CSOs were related to the person who was considered to have or to have had gambling problems.

In the Norwegian study female gender was associated with being a CSO, whereas the Swedish study indicated that males were somewhat more likely to be CSOs than females [9]. Among the Norwegian population, young age, in particular two age groups under 44 years old, were found to be positively associated with being a CSO [3]. Similarly to the Norwegian study, a Swedish population study found more CSOs in the age groups 18–24 years and 25–44 compared to other age groups [9].

Lower level of education and in particular mid-level education have been associated with being a CSO [9]. Also, divorced marital status [3,9] and separated status [9] have been associated with being a CSO. In regard to gender, single males have been found to be the most prevalent type of CSO, while being a single parent was more likely to be associated with being female. In addition, results in a clinical context have indicated that marital discord, including the threat of separation or divorce and dissatisfaction in a relationship, were relatively common among the problem gamblers’ families [2,12].

Problem-gambling prevalence has been found to be higher among Swedish male CSOs compared with females [9]. Research in a clinical context has also shown that both positive attitudes towards gambling and parental gambling involvement may be linked to a child’s own gambling behaviour [13-15]. Mother’s low education, smoking or alcohol use has been associated with young adults’ gambling behaviour, explicitly with how much they were likely to gamble or spend money on gambling [16]. All in all, how the gambler’s gambling behaviour influences the CSO’s own gambling behaviour has been studied little at the population level so far.

CSOs experienced poorer general health [3] and poorer mental health than the general population [3,9]. They also experienced less contact with the family and friends than the general population, possibly reflecting isolation and loneliness [3]. Svensson and colleagues reported that CSOs experience symptoms of depression and feelings of melancholy. Both male and female CSOs reported more arguments with someone close and female CSOs had more sick leaves from work than the general population. All of the above experiences having the potential to lead towards loneliness [9].

Previous studies in a clinical context have indicated that gambling problems within a family distress both children and the spouse. Distress includes loss of trust and security, along with a lower quality of life [17]. Parents’ gambling problems have long lasting and negative consequences for children: they have expressed feeling abandoned, rejected, neglected, emotionally deprived, angry, hurt, sad, confused, isolated or/and lonely, guilty, helpless, anxious and depressed [18-20]. Furthermore, child CSOs suffer from increased suicide rates and substance abuse [12,20]. Gamblers’ spouses often suffer from stress-related difficulties, including headaches, intestinal disorders, faintness, breathing irregularities, backaches, asthma, high blood pressure and insomnia [12,19].

Both previous population studies found alcohol and substance abuse to be associated with being a CSO [3,9]. In addition, previous studies in the clinical context indicate that children of parents who gambled had a greater risk of being involved in health threatening behaviours such as smoking, drinking, drug use and overeating [21].

To summarise, previous research implies that several socio-demographic and gambling-, health- and well-being-related factors are or may be associated with being a CSO. Previous studies have been mainly conducted from the perspective of female spouses in a clinical context. Further studies on CSOs and particularly the investigation of gender differences are needed. The aims of this study were to investigate the proportion of male and female CSOs at the population level; to investigate who the CSOs were concerned about; and to investigate sociodemographic factors, gender differences, gambling behaviour, and health and well-being among CSOs and non-CSOs.

## Methods

### Design, participants and data collection

A correlational and cross-sectional study design was used. The data were based on a population study entitled: ‘Finnish Gambling 2011’ [22,23]. A random sample of 16 000 Finns was selected from the Finnish Population Information System. Inclusion criteria were: 1) aged 15–74 years, 2) Finnish or Swedish native-language, and 3) living in mainland Finland.

A market research company Taloustutkimus Ltd was responsible for conducting the data collection [22]. A landline or mobile telephone number was available for 11 129 participants. No manual or second-hand searches were performed. In addition, Taloustutkimus Ltd contacted 4870 participants without a phone number by traditional mail to invite them to the study and asked them to leave their phone number into an answering machine. With this method an additional 120 phone numbers were resolved. The survey was described to the respondents as ‘a gambling and health survey’ [22].

In total, 11 249 Finns were approached by telephone between the 3^rd^ of October 2011 and the 14^th^ of January 2012. A total of 166 interviewers conducted structured telephone interviews. Each interviewer was trained and supervised by Taloustutkimus Ltd. In contacting potential interviews, 757 phone numbers were found to be invalid, and 1724 respondents could not be reached after a maximum of 10 attempts, while a further 4279 people refused to participate and five quit the interview after it had begun. The final number of respondents was 4484, 40% of the phone numbers that were eventually found to be operable. Data were weighted by age, gender and residency [22]. The weighted number of respondents was 4031 (population estimate 4 031 000). The Ethics committee of the National Institute of Health and Welfare approved the research protocol.

### Measurements

*Concerned significant others* (CSOs) were evaluated by inquiring: ’Have any of the following significant others had problems with gambling?’ Seven options for significant other were available (each with response options ‘yes’, ‘no’, and ‘do not know’): father, mother, sister/brother, grandparent, spouse, own child/children and close friend. A dichotomous variable was created to indicate whether the respondent had at least one significant other with gambling problems: response options ‘no’ and ‘do not know’ values were combined.

*Demographic correlates* consisted of respondent’s sex (male, female) and marital status (married or registered relationship, cohabiting, separated or divorced or widowed, single). Age was recoded into six groups (15–17, 18–24, 25–34, 35–49, 50–64, ≥65 years) and education into two groups (≤12 years, >12 years).

*Gambling-related correlates* included any past-year gambling involvement (yes, no), while the number of game types engaged in during the past year was recoded into three groups: 1) 0–2 games, 2) 3–4 games, 3) ≥5 games. Gambling problems were measured using the South Oaks Gambling Screen (SOGS) [24,25]. The SOGS was originally developed to identify lifetime pathological gamblers in the clinical context. Using a score of four or more to identify problem gambling, it demonstrated good reliability and validity, and unsurprisingly, a high correlation with DSM–III-R criteria for pathological gambling (r = 0.94); it was able to accurately classify problem gamblers from among Gamblers Anonymous members (98.1%), university students (95.3%) and hospital employees (99.3%) [24]. In our study, the Cronbach’s alpha for the SOGS was 0.92.

There has been a concern that SOGS may yield a high false positive score in population studies [26,27]. In addition, a comparison of population prevalence studies indicated that the lifetime problem-gambling prevalence (SOGS ≥3) was on average 0.44 times higher than the past-year prevalence [27]. Therefore, to accurately evaluate a current problem, a 12-month time-frame was adopted. For public health research, it was more appropriate to concentrate on problem gamblers (SOGS ≥3) than the small group of pathological gamblers. In addition, non-gamblers were separated into their own group. Age of onset of gambling was dichotomised into two groups (<18 years, ≥18 years) based on the age limit for legal gambling in Finland.

*Health- and well-being related correlates* were also investigated. General health was inquired using a question: ‘How is your general health at present?’. Five response options were dichotomised into two groups: 1) average, good or somewhat good and 2) bad or somewhat bad. Mental health was assessed using the Mental Health Inventory, which comprised five items: nervousness, the blues, jollity, calmness and happiness (MHI-5) [28]. The MHI-5 has a 6-point Likert scale (range 1–6). The total MHI-5 scores were calculated by summing up the score of each item, with sums (range 4–30) rescaled to 1–100. A score of 52 or less was used to indicate clinically significant mental health problems, as recommended by Berwick and colleagues [29]. The MHI-5 seems to be an adequate screen for some anxiety disorders (generalised anxiety disorder, panic disorder, obsessive compulsive disorders), but not others, especially phobias [30]. In our study, Cronbach’s alpha for the MHI-5 was 0.77. Loneliness was inquired using a question: ‘Do you feel lonely?’. Five response options for loneliness were dichotomised into two groups: 1) never or very rarely and 2) sometimes, often or all the time.

Smoking was inquired using the question: ‘Have you smoked during the past 12 months?’. Three response options for smoking were dichotomised into two groups: 1) daily smoking and 2) occasionally or not at all. Alcohol consumption was measured using a 3-item version of the Alcohol Use Disorders Identification Test (AUDIT-C) [31]. The AUDIT-C appears to be practical and valid screen for heavy drinking and/or active alcohol abuse or dependence [31]. Total score for the AUDIT-C was counted by summing the points (range 0–3) for each item and using the cut-off points recommended by Seppä (2010) to define risky drinking among Finnish males (≥6 points) and females (≥5 points) [32]. In our study, Cronbach’s alpha for the AUDIT-C was 0.61.

The Finnish versions of the instruments were translated in collaboration with qualified translators and an expert panel. All instruments were pilot tested (N = 30).

### Statistical analyses

The data were analysed using SPSS 21.0 software (SPSS, Inc., Chicago, IL, USA). Descriptive statistics included frequencies, percentages, means and standards deviation (SD). First, the gender proportions for CSOs were calculated (Table 1). Then, correlates were examined within genders: male CSOs were compared with male non-CSOs, and female CSOs were compared with female non-CSOs (Tables 2 & 3). Statistical significance (p) was determined by the chi-squared Test (>2 groups) and Fisher’s Exact Test (2 groups), and Binary Logistic Regression Analysis. Missing values were not replaced except while creating the CSO variable: missing values were included into the non-CSO category.

**Table 1:** The proportion of concerned significant others (CSOs) of problem gamblers

		**Gender of the CSOs**		
**Problem gambler**	**All CSOs %**	**Males %**	**Females %**	**Significance**
**1. Father**	2.0	2.0	2.0	p = 1.000
**2. Mother**	0.8	0.7	0.8	p = 0.859
**3. Sister or brother**	2.7	1.9	3.4	p = 0.004
**4. Grandparent**	1.0	1.0	1.0	p = 1.000
**5. Partner**	1.7	0.8	2.6	p ≤ 0.001
**6. Own child or children**	1.6	1.3	2.0	p = 0.106
**7. Close friend**	12.4	14.4	10.3	p ≤ 0.001
**At least one of above (numbers 1-7)**	19.3	19.8	18.7	p = 0.402
**At least one member in the family (numbers 1-6)**	8.6	6.8	10.4	p ≤ 0.001

Significance is determined by Fisher’s exact test; the data (N = 4484; males n = 2117, females n = 2367) were weighted based on gender, age and residency.

**Table 2:** Associations of the correlates among both male and female CSOs and non-CSOs

			**Males**			**Females**		
**Variables**		**All respondents**	**CSOs**	**Non-CSOs**	**Significance**	**CSOs**	**Non-CSOs**	**Significance**
		**%**	**(n = 399)**	**(n = 1616)**		**(n = 377)**	**(n = 1638)**	
			**%**	**%**		**%**	**%**	
**Sociodemographic characteristics**								
Age					Chi = 5.036, df =5, p = 0.411			Chi = 4.537, df =5, p = 0.475
	15-17 years	4.0	3.5	4.1		3.5	4.2	
	18-24 years	12.3	13.8	12.4		12.2	11.8	
	25-34 years	16.9	18.5	17.0		18.4	16.1	
	35-49 years	25.7	25.8	26.1		25.5	25.3	
	50-64	28.6	29.6	28.0		30.1	28.7	
	≥65	12.5	8.8	12.4		10.4	14.0	
Education ≤12 years		41.3	47.4	48.3	p = 0.737	35.5	34.1	p = 0.631
Married or registered relationship		48.3	42.9	48.3	p = 0.057	42.7	50.9	p = 0.004
**Gambling related correlates**								
Any past-year gambling involvement		77.9	84.0	82.7	p = 0.603	77.2	72.0	p = 0.040
Number of game types, past-year					Chi = 8.589, df =2, p = 0.014			Chi = 14.345, df =2, p ≤ 0.001
	0-2 game types	67.0	51.7	59.3		69.6	78.4	
	3-4 game types	22.1	26.5	24.3		24.1	18.3	
	≥ 5 game types	10.9	21.8	16.4		6.4	3.3	
Past-year gambling problems^1^					Chi = 23.762, df =2, p ≤ 0.001			Chi = 16.329, df =2, p ≤ 0.001
	No gambling	22.1	16.0	17.3		22.8	28.0	
	No problems (SOGS = 0-2)	75.2	75.3	79.4		74.3	71.2	
	Problem gambler (SOGS ≥ 3)	2.7	8.8	3.2		2.9	0.7	
Onset age for gambling less than 18 years		56.1	74.8	67.7	p = 0.007	51.0	40.5	p ≤ 0.001
**Perceived health and well-being**								
Bad general health^2^		3.0	5.0	2.9	p = 0.040	2.9	2.6	p = 0.721
Mental health problem^3^		3.3	5.9	2.7	p = 0.004	6.0	2.6	p = 0.003
Loneliness^4^		18.2	21.5	15.9	p = 0.009	23.9	18.4	p = 0.017
Smoking daily		17.8	24.6	20.2	p = 0.064	23.3	12.5	p ≤ 0.001
Risky alcohol consumption^5^		26.1	46.4	31.1	p ≤ 0.001	21.7	17.0	p = 0.053

^1^SOGS, the South Oaks Gambling Screen [23,24]^2^Bad or somewhat bad general health; ^3^MHI-5, the Mental Health Inventory, scaled 1–100, clinically significant problem ≤ 52; ^4^feeling sometimes, often or all the time lonely, ^5^The Alcohol Use Disorders Identification Test (AUDIT-C), score for risk consumption ≥ 5 among women and ≥ 6 among men; Significance (p) is determined by chi-squared (>2 groups) and Fisher’s exact tests (2 groups); the data (N = 4484; males n = 2117, females n = 2367) were weighted based on gender, age and residency.

**Table 3:** Multivariate models with the correlates among male and female CSOs

	**Model 1**		**Model 2**		**Model 1**		**Model 2**	
	**(n = 1759)**		**(n = 1866)**		**(n = 1863)**		**(n = 2030)**	
	**Male CSOs**^**a**^		**Male CSOs**^**a**^		**Female CSOs**^**b**^		**Female CSOs**^**b**^	
	**OR**	**95% CI**	**OR**	**95% CI**	**OR**	**95% CI**	**OR**	**95% CI**
**Socio-demographic characteristics**								
Age in years	1.00	0.99-1.01	†	†	1.00	0.90-1.01	†	†
Education ≤ 12 years	1.00	0.78-1.28	†	†	1.04	0.78-1.38	†	†
Not married or registered relationship	1.10	0.83-1.47	†	†	1.23	0.93-1.63	†	†
**Gambling related correlates**								
Past-year gambling, any type	0.89	0.59-1.34	1.04	0.74-1.50	1.27	0.88-1.84	1.51**	1.09-2.08
3+ game types gambled	1.13	0.86-1.48	†	†	1.38*	1.02-1.86	†	†
Past-year gambling problem^1^	1.89**	1.14-3.14	2.03**	1.24-3.31	2.40	0.88-6.50	3.05**	1.18-7.90
Onset age for gambling less than 18 years	1.32	0.98-1.77	†	†	1.41*	1.04-1.90	†	†
**Perceived health and well-being**								
Bad general health^2^	1.90	1.00-3.62	†	†	0.95	0.35-2.52	†	†
Mental health problem^3^	1.61	0.87-2.98	1.60	0.88-2.81	1.83	0.98-3.42	2.21**	1.24-3.93
Loneliness^4^	1.37	0.99-1.89	1.46**	1.08-1.97	1.39*	1.01-1.91	1.39*	1.03-1.89
Smoking daily	0.90	0.66-1.22	0.93	0.69-1.24	1.85***	1.32-2.57	1.97***	1.43-2.71
Risky alcohol consumption^5^	1.70***	1.31-2.21	1.78***	1.38-2.29	0.95	0.68-1.33	0.99	0.71-1.37
**Nagelkerke**	**0.052**		**0.042**		**0.057**		**0.049**	

Binary logistic regression analysis; ^a^ Reference group: male non-CSOs; ^b^ Reference group: female non-CSOs; ^1^the South Oaks Gambling Screen [23,24]; ^2^Bad or somewhat bad general health; ^3^MHI-5, the Mental Health Inventory, scaled 1–100, clinically significant problem ≤ 52; ^4^feeling sometimes, often or all the time lonely, ^5^The Alcohol Use Disorders Identification Test (AUDIT-C), score for risk consumption ≥ 5 among females and ≥ 6 among males; * < 0.05, ** < 0.01, *** < 0.001; †, not included; the data (N = 4484; males n = 2117, females n = 2367) were weighted based on gender, age and residency.

Two multivariate models with the correlates are presented (Table 3). All variables were included in Model 1 simultaneously. Different combinations of the above correlates were tested while creating Model 2. The poorest correlates were dropped. The best fitting model, Model 2, was selected, using statistical significance (p ≤ 0.05) among either males or females as the criteria for inclusion. Results of the logistic regression analyses are presented as odds ratios (OR) and their corresponding 95% confidence intervals (CI). Goodness of fit was assessed using Nagelkerke’s R^2^.

## Results

### Respondents

The sample comprised 4484 respondents with a mean age of 48.2 years (SD 16.8, range 15–74 years). One third (33.2%) of the respondents were younger than 35 years, 41.3% had an education of less than 12 years and almost half (48.3%) were married or lived in a registered relationship (Table 2). The majority (77.9%) had been involved in gambling within the past 12 months and one third (33.0%) had gambled three or more different game types. The past-year problem-gambling (SOGS ≥ 3) prevalence rate was 2.7 per cent. Three per cent of the respondents perceived their general health to be bad or somewhat bad and 3.3 per cent had significant mental health problems (MHI-5 ≤ 52). Almost one fifth (18.2%) perceived themselves as lonely (sometimes, often or all the time). 17.8 per cent smoked daily and a quarter (26.1%) used alcohol at a risky level (AUDIT-C ≥ 5 females, ≥ 6 males).

### Proportions of CSOs and who they were concerned about

Almost one fifth (19.3%) of the respondents had at least one significant other who had had a gambling problem (Table 1). There were no overall gender differences in the proportion of the CSOs. Most commonly the person with a gambling problem was a close friend (12.4%): Among male CSOs, the percentage of close friends (14.4%) was larger compared with females (10.3%; p ≤ 0.001). Further analysis was performed to evaluate the proportion of the respondents who had at least one family member (father, mother, sister/brother, grandparent, spouse, own child/children) with a gambling problem. The results showed that 8.8 per cent of the respondents (6.8% males, 10.4% females; p ≤ 0.001) had at least one family member who had had a gambling problem. Of family members, the person with a gambling problem was a sister or a brother (2.7%), a father (2.0%), a partner (1.7%) or own child/children (1.6%) of the CSO. Among female CSOs, the problem gambler was more often a partner (p ≤ 0.001) or a sister/brother (p = 0.004) compared with males.

### Bivariate analysis of the correlates

Age and education were not statistically significant correlates for the CSOs (Table 2). Marital status was statistically significantly associated with being a male CSO. However, the proportion of women who were married or lived in a registered relationship was bigger among the non-CSOs compared with the CSOs (p = 0.004). Being a CSO was statistically associated (regardless of gender) with a large number of game types gambled during the past year, past-year gambling problems (SOGS ≥ 3), the onset age of gambling less than 18 years, mental health problems and loneliness. Past-year gambling involvement (p = 0.040) and smoking daily (p ≤ 0.001) were associated with being a female CSO, while risky alcohol consumption (p ≤ 0.001) was associated with being a male CSO.

### Multivariate models with the correlates

The significance of the correlates was examined by using two models (Table 3). In Model 1, the following correlates were simultaneously included in the analysis: a) socio-demographic correlates (age as a continuous variable, ≤ 12 years of education, not married/in a registered relationship), b) gambling-related correlates (past-year gambling, ≥ 3 game types gambled, past-year gambling problems, onset age of gambling less than 18), c) health- and well-being-related correlates (bad general health, mental health problems, loneliness, smoking daily, risky alcohol consumption).

In Model 1, the odds ratio (95% CI) of being a male CSO was 1.89 (1.14–3.14) for past-year gambling problem and 1.70 (1.31–2.21) for risky alcohol consumption. Further, the odds ratio (95% CI) of being a female CSO was 1.38 (1.02–1.86) for three or more game types gambled, 1.41 (1.04–1.90) for the age of onset of gambling less than 18 years, 1.39 (1.01–1.91) for loneliness, and 1.85 (1.32–2.57) for daily smoking. The goodness of fit in Model 1 was 0.052 among males and 0.057 among females.

In Model 2, the odds ratio (95% CI) of being a male CSO was 2.03 (1.24–3.31) for past-year gambling problems, 1.46 (1.08–1.97) for loneliness and 1.78 (1.38–2.29) for risky alcohol consumption. The odds ratio (95% CI) of being a female CSO was 1.51 (1.09–2.08) for past-year gambling involvement, 3.05 (1.18–7.90) for past-year gambling problems, 2.21 (1.24–3.93) for mental health problems, 1.39 (1.03–1.89) for loneliness, and 1.97 (1.43–2.71) for daily smoking. The goodness of fit in Model 2 was 0.042 among males and 0.049 among females.

## Discussion

In 2011, the Finnish past-year problem gambling (SOGS ≥ 3) rate was 2.7% (population estimate 110 000). Based on Williams and colleagues, the standardised problem-gambling rate for Finland would be 1.5% in 2011, which would be considered average and which would be comparable also to other standardised rates seen in Sweden, Australia, Canada and the United States [27]. However, when looking at the impacts of one’s gambling problem from the perspective of the CSOs, the percentage is considerably higher. The large proportion of CSOs reflects the fact that an individual’s gambling problem has extensive impacts at the community level.

Almost a fifth (19.3%) of Finnish respondents were defined as CSOs (population estimate 786 000). The Finnish CSO prevalence is almost identical to that of the Swedish study [9]. However, the Swedish study used a more open approach in defining CSOs: they were not able to identify the relationship with the problem gambler in spite of their interest in doing so [9,33]. In our study, relationships to a problem gambler varied from close family members to close friends, excluding more distant friends, colleagues or relatives. Our results showed that the problem gambler was most typically not a family member but a close friend. In the Norwegian study, the approach was restricted to the family context and they identified only 2.0% of the population as CSOs [3]. Despite this, the proportion of CSOs was greater in Finland than in Norway, since 8.6% of Finnish respondents had a problem gambler in the family. The differences between our results and the Norwegian results may be because the instrument used in the Norwegian study required respondents to know that they had been lied to by their gambling relative and to have noticed that their relative had spent more and more money on gambling [3].

Overall, the proportion of the CSOs did not differ between men and women. However, females (10.4%) had at least one family member who had had a gambling problem statistically significantly more often than male CSOs (6.8%). This finding is consistent with the Norwegian population study, which found female gender associated with having a problem gambler in the family [3]. However, the Swedish population study’s more open approach indicated that males were somewhat more likely to be CSOs than females [9]. On the other hand, our results indicate that men had close friends who were problem gamblers more often than women. Therefore, the differences between these three population studies may be explained by different definitions of CSOs. In order to assure better comparability, the use of a coherent definition for CSO should be pursued in further research.

The proportion of female CSOs that were concerned about partners’ or sisters’ or brothers’ gambling was larger than the proportion of the male CSOs, whereas male CSOs were concerned about a close friend’s gambling more often than females. These gender differences may reflect that men are more likely to have gambling problems compared with women. Therefore, one may assume that women are more likely to be married or be the sibling of a problem gambler. Similarly, if men are more likely to be close friends with other men rather than other women, thus perhaps men are more likely to be friends with a problem gambler. Findings from two previous help-seeking CSO samples also indicate that CSOs were the intimate partner of or were in a relationship with a problem gambler [34,35]. In addition, help-seeking CSOs were mainly female [34]. Overall, the CSOs of problem gamblers encounter a great deal of general relationship and personal distress. However, it is probable that distress caused by family members’ gambling is different from distress caused by friend’s gambling [2,7,8,36,37], and they should be taken into account when planning and developing support for CSOs. Further studies evaluating these differences are needed.

Our bivariate analyses revealed that the gambling behaviour of the CSOs also paralleled the gambler’s gambling behaviour. First, the number of game types gambled, the past-year gambling problems (SOGS ≥ 3) and the onset age of gambling less than 18 years were associated with being a CSO for both genders. Furthermore, multivariate analyses revealed that female CSOs had two statistically significant gambling-related variables as underlying factors that may cause distress in their lives: their own gambling problem and past-year gambling. In all analyses, any past-year gambling involvement was statistically significantly associated with being a female CSO, but not being a male CSO. Further studies are needed to confirm and explore this finding.

CSOs reported impairments in several aspects of perceived health and well-being correlates: for both genders, mental health problems were significantly associated with being a CSO. This finding was in line with the Swedish study, which also found substance abuse clearly associated with being a CSO [9]. In our multivariate analyses, risky alcohol consumption was statistically significantly associated with being a male CSO, while daily smoking was statistically significantly associated with being a female CSO. In fact, CSO’s daily smoking was the only correlate where the 95% CI did not overlap for males and females (Table 3). Therefore, the clearest gender difference that might have practical importance [38] was seen in smoking. This is an intriguing finding, since smoking is also strongly associated with female gamblers: women who smoke are 14 times more likely to be pathological gamblers than non-smoking women, whereas for males the corresponding figure is five [39].

Overall, comorbid mental health problems and substance abuse, including alcohol and nicotine, are clearly associated with the severity of gambling problems [40,41]. In the clinical context, it has been proposed that smoking may enhance the gambling experience or serve as a cue for gambling among problem gamblers [39,42]. Our results indicate that similar correlates are also associated with being a CSO. Further research on CSOs should seek to establish whether it is because they live in the same environment and most likely share similar lifestyles, as has been suggested by Etcheverry and colleagues [43], or whether it is a coping method for different types of life stress.

Since there are obvious personal and familial costs associated with problem gambling, it is important to acknowledge the specific needs of CSOs whilst planning support and treatments for problem gamblers. Recovering gamblers have indeed reported that family members play an assistive role in their recovery process and that CSOs are naturally motivated to work on their own situation as well as helping the gambler to recover [2,11,42,44,45]. Reports from gambling helplines also show that CSOs request assistance and support [46,47]. The Finnish gambling helpline Peluuri provides support, information and consultation services via telephone, short message service and internet for problem gamblers and their CSOs. In 2012, 34% of the Peluuri helpline calls came from CSOs, and during 2013, CSOs reported experiencing increasingly more social- and health-related harms due to the gambling of their significant other [48,49].

The representativeness of this population study is limited by attrition. However, the data were weighted based on age, gender and residency in order to enhance sample representativeness [22]. A power analysis was not calculated when determining the sample size. The sampling frame was drawn from both landline phones and mobile phones, but the relevance of the use of the dual-frame was not analysed. However, Jackson and colleagues (2013) have recommended the use of a dual-frame sampling methodology, since a traditional landline sampling has proven to impair the sample representativeness [50].

Due to the cross-sectional design, conclusions about the causality of the correlates and CSOs are not possible. In addition, our study did not look comprehensively at the different types of gambling harms that CSOs experienced. For example, Svensson and colleagues (2013) noticed that both male and female CSOs had more financial difficulties than other non-CSOs. The CSOs had lent money to someone who they thought or knew would use it to gamble or pay bills and they were more often exposed to violence during the past year than other people [9]. Previous results from clinical contexts have also indicated that emotional and physical abuse is associated with problem gambling [5-7,35]. Thus, it would be important to include these topics in further research. In particular, it would be useful to clarify the consequences of having a problem gambler as a significant other, as well as to study the help-seeking of CSOs in more depth.

Although the CSO’s gambling behaviour was assessed with a validated instrument, the gambling problems of their significant other were based on the CSOs own perceptions and were not assessed with a validated instrument or diagnostic interview. In this study, the concern mainly reflects the potential existence of the gambling problems of the significant others without any evaluation of the amount or type of concern. Additionally, the verb tense used to assess this was ‘*has had* problems’. With this type of wording, the prevalence of problem gambling as assessed by CSOs should be considered as ‘lifetime prevalence’ rather than ‘past-year prevalence’.

The past-year gambling problems, the alcohol consumption and mental health problems of respondents were evaluated using previously validated instruments [23-27]. However, with the SOGS, excessive weight is given to items concerned with borrowing money, with nearly half of the 20 equally weighted items dealing with sources for funding gambling. The SOGS has also been criticized for not being sufficiently sensitive to slot machine-related problems or to gambling problems in women [26,51]. This is notable in Finland where slot machines are among the three most popular game types and women’s gambling prevalence has increased in 2007 and 2011 [22,52].

Finally, previous population-based research on CSOs is rare and the existing theoretical or empirical knowledge on CSOs are limited. Due to the exploratory aims of this study, a large number of statistical tests were undertaken without corrections being made for multiple comparisons. Therefore, it is expected that some of the findings of significant relationships in this manuscript may be incorrect and further research is needed to test the specific hypotheses arising here so as to confirm the existence of these specific relationships. This study mainly offers valuable suggestive information and recommendations for further research.

## Conclusions

This study confirmed that gambling problems broadly affect people close to problem gamblers and that CSOs experience cumulative problems such as their own risky gambling behaviour, health problems and other addictive disorders. The clearest gender difference was seen in smoking by CSOs. With the acknowledged correlates in mind, a better recognition and understanding is needed in order to establish efficient and targeted support and services for CSOs. It is vital to identify and support CSOs both for their own sake and for the sake of the problem gambler they are close to. However, more information is needed in order to build up efficient, targeted and gender-specific support and services for CSOs.

## Competing interest

The authors declare that they have no competing interest.

## Authors’ contributions

AS, SC, HA and TL were responsible for the study conception and design; AS performed the analysis; AS and SC were responsible for the interpretation of the data and manuscript preparation; AS, SC, HA and TL made critical revisions to the paper for important intellectual content; AS, SC, HA and TL read and approved the final version.

## Pre-publication history

The pre-publication history for this paper can be accessed here:

http://www.biomedcentral.com/1471-2458/14/398/prepub
